# Podoplanin increases the migration of human fibroblasts and affects the endothelial cell network formation: A possible role for cancer-associated fibroblasts in breast cancer progression

**DOI:** 10.1371/journal.pone.0184970

**Published:** 2017-09-22

**Authors:** Jaroslaw Suchanski, Anna Tejchman, Maciej Zacharski, Aleksandra Piotrowska, Jedrzej Grzegrzolka, Grzegorz Chodaczek, Katarzyna Nowinska, Janusz Rys, Piotr Dziegiel, Claudine Kieda, Maciej Ugorski

**Affiliations:** 1 Department of Biochemistry and Molecular Biology, Faculty of Veterinary Medicine, Wroclaw University of Environmental and Life Sciences, Wroclaw, Poland; 2 Laboratory of Glycobiology, Ludwik Hirszfeld Institute of Immunology and Experimental Therapy, Polish Academy of Sciences, Wroclaw, Poland; 3 Centre for Molecular Biophysics, Cell Recognition and Glycobiology, UPR4301-CNRS, Orléans, France; 4 Department of Histology and Embryology, Wroclaw Medical University, Wroclaw, Poland; 5 EIT+ Wroclaw Research Center, Wroclaw, Poland; 6 Department of Tumor Pathology, Centre of Oncology, Maria Sklodowska-Curie Memorial Institute Cracow Branch, Cracow, Poland; 7 Department of Physiotherapy, Wroclaw University School of Physical Education, Wroclaw, Poland; 8 Military Medical Institute, Warsaw, Poland; University of South Alabama Mitchell Cancer Institute, UNITED STATES

## Abstract

In our previous studies we showed that in breast cancer podoplanin-positive cancer-associated fibroblasts correlated positively with tumor size, grade of malignancy, lymph node metastasis, lymphovascular invasion and poor patients’ outcome. Therefore, the present study was undertaken to assess if podoplanin expressed by fibroblasts can affect malignancy-associated properties of breast cancer cells. Human fibroblastic cell lines (MSU1.1 and Hs 578Bst) overexpressing podoplanin and control fibroblasts were co-cultured with breast cancer MDA-MB-231 and MCF7 cells and the impact of podoplanin expressed by fibroblasts on migration and invasiveness of breast cancer cells were studied in vitro. Migratory and invasive properties of breast cancer cells were not affected by the presence of podoplanin on the surface of fibroblasts. However, ectopic expression of podoplanin highly increases the migration of MSU1.1 and Hs 578Bst fibroblasts. The present study also revealed for the first time, that podoplanin expression affects the formation of pseudo tubes by endothelial cells. When human HSkMEC cells were co-cultured with podoplanin-rich fibroblasts the endothelial cell capillary-like network was characterized by significantly lower numbers of nodes and meshes than in co-cultures of endothelial cells with podoplanin-negative fibroblasts. The question remains as to how our experimental data can be correlated with previous clinical data showing an association between the presence of podoplanin-positive cancer-associated fibroblasts and progression of breast cancer. Therefore, we propose that expression of podoplanin by fibroblasts facilitates their movement into the tumor stroma, which creates a favorable microenvironment for tumor progression by increasing the number of cancer-associated fibroblasts, which produce numerous factors affecting proliferation, survival and invasion of cancer cells. In accordance with this, the present study revealed for the first time, that such podoplanin-mediated effects can affect tube formation by endothelial cells and participate in their pathological properties in the tumor context. Our experimental data were supported by clinical studies. First, when IDC and DCIS were analyzed by immunohistochemistry according to the presence of podoplanin-expressing cells, the numbers of cancer-associated fibroblasts with high expression of this glycoprotein were significantly higher in IDC than in DCIS cases. Second, using immunofluorescence, the co-localization of PDPN-positive CAFs with blood vessels stained with antibody directed against CD34 was observed in tumor stroma of IDC samples.

## Introduction

Podoplanin (PDPN) is a highly *O*-glycosylated, transmembrane mucin-type glycoprotein, which is widely distributed on the surface of various types of normal animal cells [1]. However, during malignant transformation, the expression of PDPN undergoes profound quantitative and qualitative changes [2,3]. Neoexpression or highly increased expression of this mucin-type protein on tumor cells can be observed in different human malignancies, such as squamous cell carcinomas of lung, skin, head and neck and animal experimental tumors [3]. The role of PDPN in carcinogenesis and tumor progression has been studied intensively. It has been shown that this glycoprotein is an adhesive molecule involved in the aggregation of tumor cells and platelets since the specific *O*-glycan carried by the extracellular domain of podoplanin is the ligand for the lectin-like protein called C-type lectin like receptor 2 (CLEC-2) expressed by platelets [4,5]. Such tumor cell-platelet aggregates play an important role in metastatic cascade [6,7]. This transmembrane glycoprotein also promotes cancer cell migration and invasion, since the podoplanin cytoplasmic tail interacts with ezrin and/or moesin, members of the ERM protein family, to activate RhoA and promote epithelial-mesenchymal transition (EMT) [8–11].

Interestingly, podoplanin is absent in tumor cells from several invasive adenocarcinomas such as breast, lung or pancreas. Instead, in such malignancies, this glycoprotein is highly expressed in cancer associated fibroblasts (CAFs) [3], which are the components of tumor stroma [12]. A substantial amount of clinical data shows that the presence of PDPN-expressing CAFs correlates with poor patients’ prognosis in such cancers [13–22]. CAFs promote cancer progression as they secrete various growth factors, chemokines and cytokines, which stimulate proliferation rate, migration and invasiveness of tumor cells as well as angio- and lymphogenesis [12,23–25]. Very little is known about the role of podoplanin expressed by CAFs, its biological properties or its function as a tumor promoter. It was shown using nude mice model, that human vascular adventitial fibroblasts (hVAF) with high PDPN expression enhanced tumor progression of human lung adenocarcinoma cells more than human lung tissue-derived fibroblasts (hLF) with low expression of this glycoprotein [21]. Further study, using a mutated form of podoplanin lacking an intracellular domain, revealed that the tumor-promoting activity of PDPN is associated with increased activity of RhoA [26]. It was proposed that enhanced RhoA activity facilitates PDPN-positive-fibroblasts to create a microenvironment promoting cancer growth. Using a newly developed collagen invasion assay, Neri et al. [27] found that CAFs with high expression of podoplanin invaded the matrix to a greater extent, and more importantly, with more cancer cells traveling within the “tracks” created by the CAFs, than with CAFs with low-expression of this glycoprotein. These data were confirmed by *in vivo* studies. When mice were injected intravenously with CAFs and tumor cells simultaneously, it was found that PDPN-high CAFs invaded in larger amounts and promoted cancer cell invasion into the lung parenchyma, more than with PDPN-low CAFs. High expression of podoplanin was also found in some CAFs from invasive ductal carcinoma of the pancreas [20]. When pancreatic cancer cells were co-cultured with fibroblasts having high podoplanin expression, their motility and invasiveness were increased in comparison to CAFs with low expression of the PDPN. However, the suppression of PDPN in such cells by siRNA did not affect the biological properties of tumor cells, which suggests that this glycoprotein is not directly responsible for their migration and invasiveness. Overall, the role of podoplanin expressed by CAFs in cancer progression remains ambiguous and inconsistent.

In our previous studies we showed that in breast cancer PDPN-positive CAFs correlated positively with tumor size, grade of malignancy, lymph node metastasis, lymphovascular invasion and poor patients’ outcome [14]. Therefore, in the present study, the impact of podoplanin expression in fibroblasts on biological properties of breast cancer and endothelial cells was studied. It was shown that podoplanin present on the surface of fibroblasts does not directly affect the malignant properties of breast cancer cells, but increases their motility, facilitating in this way the movement of fibroblasts into tumor stroma. Interestingly, PDPN-rich fibroblasts interact with endothelial cells and affect pseudo tube formation.

## Materials and methods

The study protocol was approved by the Bioethical Committee of the Wroclaw Medical University (reference number: KB-461/2015), and all participants gave their written informed consent to participate.

### Cell lines and tissue specimens

The following human fibroblastic cell lines were used in this study: MSU1.1 (Centre National de la Recherche Scientifique, Orleans, France) [28] and Hs578Bst purchased from the American Type Culture Collection (ATCC, Manassas, VA). MSU1.1 cells derived from normal human skin and Hs578Bst cells derived from normal breast tissue peripheral to ductal carcinoma strongly express vimentin and S100 Calcium Binding Protein A4 (S100A4/FSP1), and weakly α-SMA (IHC). Human breast cancer cell lines: MDA-MB-231 and MCF7 were obtained from ATCC. Cells were cultured in DMEM supplemented with 10% fetal calf serum (FCS; Invitrogen, Carlsbad, CA, USA), 2 mM L-glutamine, 100 U/ml streptomycin, and 0.1 mg/ml penicillin (complete DMEM). Human skin microvascular endothelial HSkMEC cells, previously described [29,30] (CNRS patent No 99–16169), were cultured in OptiMEM-1 with Glutamax-1 (Invitrogen, Cergy Pontoise, France) supplemented with 0.5 μg/ml fungizone, 40 μg/ml gentamycin and 2% heat inactivated MycoPlex FCS (PAA, Les Mureaux, France). LentiX 293T cells were purchased from Clontech Laboratories (Terra Bella Avenue, Mountain View, CA, USA) and cultured in αMEM supplemented with 10% FCS (Invitrogen), 2 mM L-glutamine, 100 U/ml streptomycin, and 0.1 mg/ml penicillin (complete αMEM).

Tumor samples and clinicopathological data of patients with invasive ductal carcinoma (IDC) and ductal carcinoma in situ (DCIS) were obtained from the Department of Tumor Pathology, Center of Oncology Maria Sklodowska-Curie Memorial Institute, Cracow. The material for IHC investigations involved 27 paraffin blocks of DCIS and 95 paraffin blocks of IDC. Patients were diagnosed from 2000 to 2007, and were aged between 27 and 82 years (mean age: 56.52 years). Clinical and pathological traits of the patients are presented inTable 1. Histopathological evaluation of the hematoxylin and eosin (H&E) stained slides was used to determine the type and the malignancy grade of the tumors (G) according to WHO criteria [31].

**Table 1:** Patients and tumor characteristics of IDC cases.

Mean age in years (range)	56,52 ± 11,39 (27–82)	
Parameters	Number	%
**Age**		
<57	42	44.21
≥57	52	54.74
Lack of data	1	1.05
**Menopausal status**		
Pre	34	35.79
Post	60	63.16
Lack of data	1	1.05
**Grade of malignancy**		
G1	5	5.26
G2	54	56.84
G3	36	37.89
**pT**		
T1	44	46.32
T2	49	51.58
T3	2	2.11
T4	0	0
**pN**		
pN0	20	21.05
pN1, pN2, pN3	75	78.95
**ER**		
Positive	59	62.11
Negative	34	35.79
Lack of data	2	2.11
**PR**		
Positive	53	55.79
Negative	40	42.11
Lack of data	2	2.11
**HER-2 by IHC and FISH**		
Positive	20	21.05
Negative	72	75.79
Lack of data	3	3.16
**Ki-67**		
<25%	30	31.58
≥25%	65	68.42

### Construction of vectors, virus production, and transductions

For generation of the PDPN expressing vector, first the IRES sequence derived from the pWP1 vector (kindly provided by Dr. D. Trono, École Polytechnique Fédérale de Lausanne, Switzerland) and puromycin N-acetyl-transferase (PAC) cDNA from a pPUR vector (Clontech) were cloned into pRRL-cPPT-CMV-X2-PRE-SIN vector (Dr D. Trono, École Polytechnique Fédérale de Lausanne, Switzerland). The resulting construct was named pRRL-CMV-IRES-PURO. To obtain human PDPN cDNA sequence, total RNA was isolated from HEK 293T cells and cDNA library was generated using Total RNA Mini Kit (A&A Biotechnology, Gdynia, Poland). PDPN cDNA was amplified by PCR with primers: 5’CGAATTCATGTGGAAGGTGTCAGCTC3’ and 5’GAACGCGTTTAGGGCGA GTACCTT3’ containing, respectively, restriction sites for EcoRI and MluI and cloned into pRRL-CMV-IRES-PURO vector. This vector was named pRRL-CMV-PDPN-IRES-PURO.

For lentivirus production packaging LentiX 293T cells were co-transfected at 50–60% confluence with 20 μg of expression or control vectors, 10 μg pMDL-g/p-RRE, 5 μg pRSV-REV, 5 μg pMk-VSVG (kindly provided by Dr. D. Trono, École Polytechnique Fédérale de Lausanne, Switzerland) using polyethylenimine (Sigma-Aldrich, Buchs, Switzerland). Culture supernatants containing virus particles were collected 48 hours after transfection and clarified through a 0.45 μm pore size filter (Millipore, Billerica, MA, USA). Immediately afterwards, the virus-containing supernatant was concentrated 100× on an Amicon Ultra-15K:100.000 (Millipore).

Cells (5x10^4^) were infected with concentrated virus stock by centrifugation (2460×g) at 23°C for 2.5 h. After overnight incubation, the medium was replaced with fresh complete DMEM.

### siRNA transfections

Transfections with 3 different siRNA against ezrin (Santa Cruz Biotechnology, Santa Cruz, CA, USA) were performed according to Fast-Forward Protocol Reverse-Transfection Protocol (Qiagen, Hilden, Germany). Cells were incubated with specific siRNA or non-silencing siRNA with no homology to any known mammalian gene. Shortly before transfection, cells (2x10^5^/well) were seeded in 6-well plates in 2 ml of DMEM culture medium containing 10% FCS. The transfection reagent was prepared by diluting 375 ng/6 μl of appropriate siRNA and 12 μl of HiPerfect Reagent (Qiagen) in 400 μl of DMEM without FCS. Cells were incubated with transfection complexes under their normal growth conditions for 48 h, harvested by trypsinization and subjected to further experiments.

### RhoA activity assay

RhoA activity was measured by a pull-down assay according to the manufacturer’s instructions for a RhoA Activation Assay Kit (Cytoskeleton, Denver, USA). Cells were detached from the plates by scraping with a cell scraper and solubilized on ice for 20 min in lysis buffer (125 mM HEPES, pH 7.5, 750 mM NaCl, 5% NP-40, 50 mM MgCl_2_, 5 mM EDTA, 10% Glycerol). GTP-bound RhoA was then immunoprecipitated from lysate with Rho-binding domain (RBD) of Rhotekin bound to agarose beads. The beads were washed and RhoA was analyzed by Western blot analysis using RhoA-specific monoclonal antibody. The lysate (20 μg) was also probed for RhoA and β-actin to quantify protein.

### SDS PAGE and Western blotting

Cells were detached using a plastic cell scraper and solubilized in 100 μl of lysis buffer [20 mM Tris-HCl, pH 8.0, 150 mM NaCl, containing 1 mM EDTA, 0.5% NP40, and 1 mM phenylmethylsulfonyl fluoride (Roth, Karlsruhe, Germany)]. The soluble proteins were quantified by the bicinchoninic acid assay (Sigma). Membrane proteins were isolated using Mem-PER Plus membrane Protein Extraction Kit (Pierce, Rockford, IL, USA) according to the manufacturer’s protocol. Briefly, cells scraped off from the bottom of the culture plate were washed twice with PBS and permeabilized with supplied detergent to release the cytosolic proteins. Then, a second detergent was used to solubilize membrane proteins.

Cell lysates or membrane proteins were subjected to vertical sodium dodecyl sulfate polyacrylamide gel electrophoresis (SDS-PAGE) (12%) according to Laemmli (1970). Separated proteins were transferred to nitrocellulose (Schleicher-Schuel, Dassel, Germany) and blotted proteins were incubated with primary antibodies. For the detection of podoplanin, the membranes were incubated with rabbit polyclonal antibodies against human podoplanin (Santa Cruz Biotechnology). For the detection of ezrin and phospho-ezrin, membranes were incubated, respectively, with rabbit polyclonal antibodies against human ezrin (Proteintech, Rosemont, USA) and Phospho-Ezrin (Tyr567) (antibodies-online, Atlanta, USA). After washing, the blots were incubated with primary antibodies overnight at 4°C, and then with horseradish peroxidase (HRP)-conjugated goat polyclonal antibodies directed against rabbit or murine immunoglobulins at room temperature (RT) for 1 hour (Dako, Carpinteria, CA, USA). The quantifications of cytoplasmic proteins were based on β-actin expression determined by utilizing anti-human β-actin murine monoclonal antibody (Abcam, Cambridge, UK).

### Real-time PCR assay

Total RNA was purified from 1x10^6^ cells using the Total RNA Mini Kit (A&A Biotechnology, Gdynia, Poland) according to the manufacturer's instructions. Reverse transcriptase reaction was performed using iScript cDNA Synthesis Kit (Biorad, Hercules, CA, USA). The relative expression level of PDPN mRNA was determined by real-time PCR assay (qPCR) with EvaGreen SuperMix (Biorad) according to the manufacturer’s protocol, using iQ5 Optical System (Biorad). The sequences of primers used in the current study were as follows: PDPN—5’-TTGACAACTCTGGTGGCAACA-3’ (forward) and 5’-GCTGTGGCGCTTGGACTT-3’ (reverse); β-actin—5’AGAAAATCTGGCACCACACC3’ (forward) and 5’AGAGGCGTACAGGGATAGCA3’ (reverse). The qPCR conditions were as follows: initial denaturation at 94°C for 30 s, then 35 cycles of denaturation at 94°C for 10 s, annealing and elongation at 60°C for 15 s. All samples were analyzed in triplicate. *ACTB* gene expression was used as an internal control. Gene expression was calculated using the ΔΔCt method.

The mRNA expression of MMP1, MMP2, MMP3, MMP9, TIMP1, VEGF-A, VE-cadh, ANGPT-1, ANGPT-2, FGF-1 was determined by qPCR with 7500 Real-Time PCR System and TaqMan Gene Expression Master Mix (Applied Biosystems, Waltham, MA, USA), according to the manufacturer’s protocols. β-actin was used as a reference gene. All reactions were performed in triplicate under the following conditions: activation of polymerase at 50°C for 2 min, initial denaturation at 94°C for 10 min, followed by 40 cycles of denaturation at 94°C for 15 s, and annealing with elongation at 60°C for 1 min. The relative expression of the studied genes was calculated with the ΔΔCt method.

### Analysis of protein distribution with confocal microscopy

To analyze cellular distribution of ezrin and Rho-GDP dissociation inhibitor (Rho-GDI), rabbit polyclonal anti-Ezrin antibodies (1:3200, Proteintech) and mouse monoclonal anti-Rho-GDI antibody (1:50, Santa Cruz Biotechnology) were used, and followed by staining with secondary antibodies labeled with TRITC and FITC, respectively. Prior to the immunostaining, plasma membrane was stained with a conjugate of wheat germ agglutinin (WGA) and Alexa Fluor 647 and cytoplasm was labeled with CellTrace Violet (CTV) dye (both reagents from Invitrogen, 20 min incubation of live cells at 37°C). Cells were imaged on a Cell Observer SD confocal system (Zeiss, Oberkochen, Germany) equipped with EMCCD QImagingRolera EM-C^2^ camera using 1x1 binning. The imaging was performed sequentially using 405 nm, 488 nm, 561 and 635 nm laser lines with quadruple dichroic mirror 405+488+561+640 and 450/50, 520/35, 600/52 and 690/50 emission filters. From each experimental condition, three to five random areas were chosen and Z stacks were taken with a 0.5 μm step using 40–63× oil objectives (0.167 μm and 0.106 μm per pixel, respectively). The same acquisition settings were applied to all conditions. For analysis, cells’ equatorial optical section was chosen and pre-processed in the ImageJ software using *Subtract Background* function with rolling ball radius of 50 pixels. The WGA and CTV channels were thresholded and binarized in order to create mask channels showing plasma membrane and cytoplasm, respectively. Any potential overlap between plasma membrane and cytoplasm was removed with *Image Calculator* function–subtraction of overlapping structures. *Analyze Particles* function was applied to the WGA and CTV masks and then identified regions were transferred onto ezrin and Rho-GDI channels to calculate fluorescence mean intensities within membrane and cytoplasm followed by quantification of their ratio.

### Invasion and migration assays

Invasion and migration assays were performed using, respectively, BioCoat Matrigel^TM^ Invasion Chamber (pore size of 8 μm) and HTS FluoroBlok Membrane Insert (pore size of 8 μm) for 24-well Plate (Corning, MA, USA). The cells were collected by trypsinization, resuspended in 1 ml PBS at a density of 10^6^/ml and labeled with 5 μl DiO (Invitrogen, Carlsbad, CA, USA) at 37°C for 30 min. For invasion assay, the plate was removed from –20°C storage and allowed to come to room temperature. 0.2 ml of warm (37°C) DMEM was added to the interior of the insert wells and left for 2 hours at 37°C, 5% CO_2_ to rehydrate. After rehydration, PBS was removed from the insert well. Fluorescent labeled cells were washed three times with warmed, serum-free DMEM and 0.2 ml of cell suspension (5x10^4^) was added to the apical chambers, while 0.6 ml of DMEM containing 10% FBS as a chemoattractant was added to the basal chamber. For co-cultures, 5x10^4^/200 μl fluorescent labeled fibroblastic or cancer cells and 5x10^4^/200 μl of unlabeled, respectively, cancer or fibroblastic cells were seeded together in the upper chambers. For invasion assay, the cells were incubated for 24 hours at 37°C, 5% CO_2_. After incubation, non-invading cells were removed from the upper surface of the Matrigel^TM^ membrane by scrubbing with a cotton tipped swab. The fluorescence of the invaded cells was read at wavelengths of 485 nm and 538 nm (for excitation and emission, respectively) on a bottom-reading fluorescent microplate reader (Fluoroscan Ascent FL, Thermo Scientific, Waltham, MA, USA). The migration assay was performed utilizing essentially the same protocol, but without rehydration, and fluorescence was measured every 4 hours. The assays were performed in triplicates, independently three times.

### Wound-healing assay

To measure the migratory properties of fibroblasts and breast cancer cells, the ibidi Culture-Insert system (ibidi GmbH Munich, Germany) with two chambers to separate different cell types was used. The Culture Inserts were placed in the compartments of a 24-well plate, 2.8x10^4^ cells were seeded into each chamber and grown to confluence in OptiMEM containing 2% FCS for 24 h. Then the silicon insert was removed with tweezers and a cell–free gap (500 μm ± 50 μm) was generated. Pictures (three/well) of wound closure were taken every hour for 48 hours, and cell migration was assessed using ImageJ software (National Institute of Health, available from http://rsb.info.nih.gov/ij; access date: 01 Oct 2015).

### Matrigel^TM^ pseudo tube formation assay with skin microvascular endothelial HSkMEC cells

Fibroblasts were cultured in 10 cm^2^ dishes in complete OPTI-MEM media one week prior to the experiment. Fibroblast cells and HSkMEC cells were labeled, respectively, with fluorescent dye DiO (Invitrogen) and fluorescent dye DiD (Invitrogen) according to the manufacturer's protocol. For angiogenic assay, HSkMEC cells were used at a density of 1x10^5^ cell/ml and MSU1.1 NC or MSU1.1 PDPN fibroblasts at a density of 0.5x10^5^ cell/ml. For the purpose of the assay, the wells of a 96-well plate (Sarstedt, Germany) were covered with 40 μl of Matrigel^TM^ Growth Factor Reduced GFR (Corning) and left at 37°C for 30 min. Then 100 μl of each cell suspension (fibroblasts or endothelial cells) or both cell suspensions (fibroblast-endothelial co-cultures) were added to the wells and cells were grown for 24 h at 37°C. Endothelial tube formation was analyzed by taking photographs every half hour with videomicroscope Zeiss Axiovert 200M and AxioVision Software (Zeiss, Germany). A toolset of Image J software was used for analysis of cellular networks [32]. The number of nodes and number of meshes were quantified on fluorescence images of HSkMEC, MSU1.1 NC and MSU1.1 PDPN cells after 12 hours. A node was defined as pixels that have at least 3 neighbors, corresponding to a bifurcation. The meshes were defined as areas enclosed by the segments. Quantitative statistical co-localization on two-color images using a software tool to calculate the Pearson and Spearman correlation coefficients (‘Pearson–Spearman correlation colocalization’ ImageJ plug-in) across regions of interest within the image was used. A quantitative estimate of the amount of co-localization in the images was given as the result.

### Immunohistochemistry (IHC)

The 4-μm-thick sections were deparaffinized and antigen retrieval was performed in Target Retrieval Solution (pH 9, 97˚C, 20 min). Then, the sections were rinsed in TBS/0.05% Tween buffer for 3 min. Endogenous peroxidase activity was blocked using EnVision FLEX Peroxidase-Blocking Reagent. In the next steps sections were washed in TBS/0.05% Tween buffer and incubated with a primary antibody directed against podoplanin (monoclonal mouse anti-human D2-40, clone D2-40, #IS072, Ready-to-Use, (Dako) in accordance with the manufacturer’s instructions in Link48 Autostainer. Following this, EnVision FLEX/HRP secondary antibodies were applied (20 min at room temperature) with 3,3’-diaminobenzidine (DAB) utilized as the peroxidase substrate. Finally, the sections were counterstained with Mayer’s haematoxylin, dehydrated in alcohols (70%, 96% and 99.8%) and xylene and mounted in SUB-X Mounting Medium. The antibodies, reagents and equipment were obtained from Dako. Immunostained sections were assessed under magnifications of ×100 and ×200 and digitally imaged for evaluation of podoplanin expression in stromal CAFs. Expression of podoplanin was also evaluated in stromal cells at the tumor margin. Only those slides were evaluated where the whole margin of tumor was evident (29 cases). The evaluation of IHC expression was semiquantitative and has taken into account the percentage of podoplanin positive tumor marigin: 0% - 0 pts; 1–5% - 1 pts, 6–15% - 2 pts, 16–25% - 3 pts, 26–50% - 4 pts, >50% - 5 pts. For the evaluation of podoplanin expression intensity the semiquantitative immunoreactive score (IRS) of Remmele and Stegner [33] was used. This method takes into account both the intensity of the staining (color) reaction and the percentage of positive cells.

### Immunofluorescence (IF)

For IF reaction, paraffin blocks were cut in sections 4 μm thick. Deparaffinisation, hydration and heat induced epitope demasking were carried out in Dako PT Link apparatus (Dako) in Target Retrieval Solution (Dako) (pH 9, 97˚C, 20 min). For the evaluation of podoplanin and CD34 co-expression in IDC immunofluorescence reactions were prepared with the use of specific anti-human monoclonal rabbit anti-CD34 antibody (dilution 1:400; clone EP373Y; code no. ab81289, Abcam) and mouse anti-PDPN antibody (dilution 1:50; clone D2-40, code no. M3619, Dako). Overnight incubation with primary antibodies was made at 4˚C. Slides were rinsed with PBS and were incubated for 1 h in room temperature with donkey anti-mouse Alexa-Fluor 594 conjugated (code no. R37115, Invitrogen) and donkey anti-rabbit Alexa-Fluor 488 secondary antibody (code no. A-21206, Invitrogen) diluted 1:2000 in the Antibody diluent with background-reducing component (Dako). Preparations were mounted using ProLong Gold Mounting Medium (Invitrogen), with DAPI for DNA visualisation. BX51 microscope (Olympus, Tokyo, Japan) coupled with CellF software (Olympus) was used for observations at x200 magnification.

### Statistical analysis

Prism 5.0 software (GraphPad, La Jolla, CA, USA) was utilized to analyze the data. The Mann-Whitney test was used to compare the groups of data that did not meet the assumptions of the parametric test. Two-way ANOVA with the Bonferroni multiple comparison test was used to analyze the differences between the analyzed cell lines. In all analyzes, the results were considered statistically significant when *p* < 0.05. Image J software (open source) was used to calculate the amount of co-localization in the selected images. The Pearson and Spearman correlation coefficient was measured and the amount of co-localization in the images was given as the result.

## Results

### Generation of human fibroblastic cell lines overexpressing podoplanin

To obtain cellular models representing gain-of-function phenotype, human fibroblastic cell lines (MSU1.1 and Hs578Bst) were analyzed by Western blotting for the presence of podoplanin. Using polyclonal antibodies directed against podoplanin, this glycoprotein was not detected in lysates of either cell line. Therefore, the cells were transduced with pRRL-CMV-PDPN-IRES-PURO expression vector and the resulting cell populations resistant to puromycin were screened for the presence of this mucin-type protein on the mRNA and protein levels [34] **(S1A and S1B Fig)**. The generated cells with high expression of podoplanin were named MSU1.1 PDPN and Hs578 PDPN. In the lysates of transduced cells, rabbit polyclonal antibodies bound to three protein bands with apparent molecular masses corresponding to 36 kDa, 23 kDa and 17 kDa, which agrees with data obtained by others [8]. However, in the purified membrane fraction, only one protein band with the highest molecular mass was found, which indicates that two other bands represent glycoforms of podoplanin that are not fully processed (**S1C Fig**). Control MSU1.1 NC and Hs578 NC with no expression of podoplanin were transduced with empty pRRL-CMV-IRES-PURO vector.

### Podoplanin expressed by fibroblasts does not affect the migration and invasiveness of breast cancer cell lines but it increases the motility of fibroblastic cell lines

**Fig 1:**
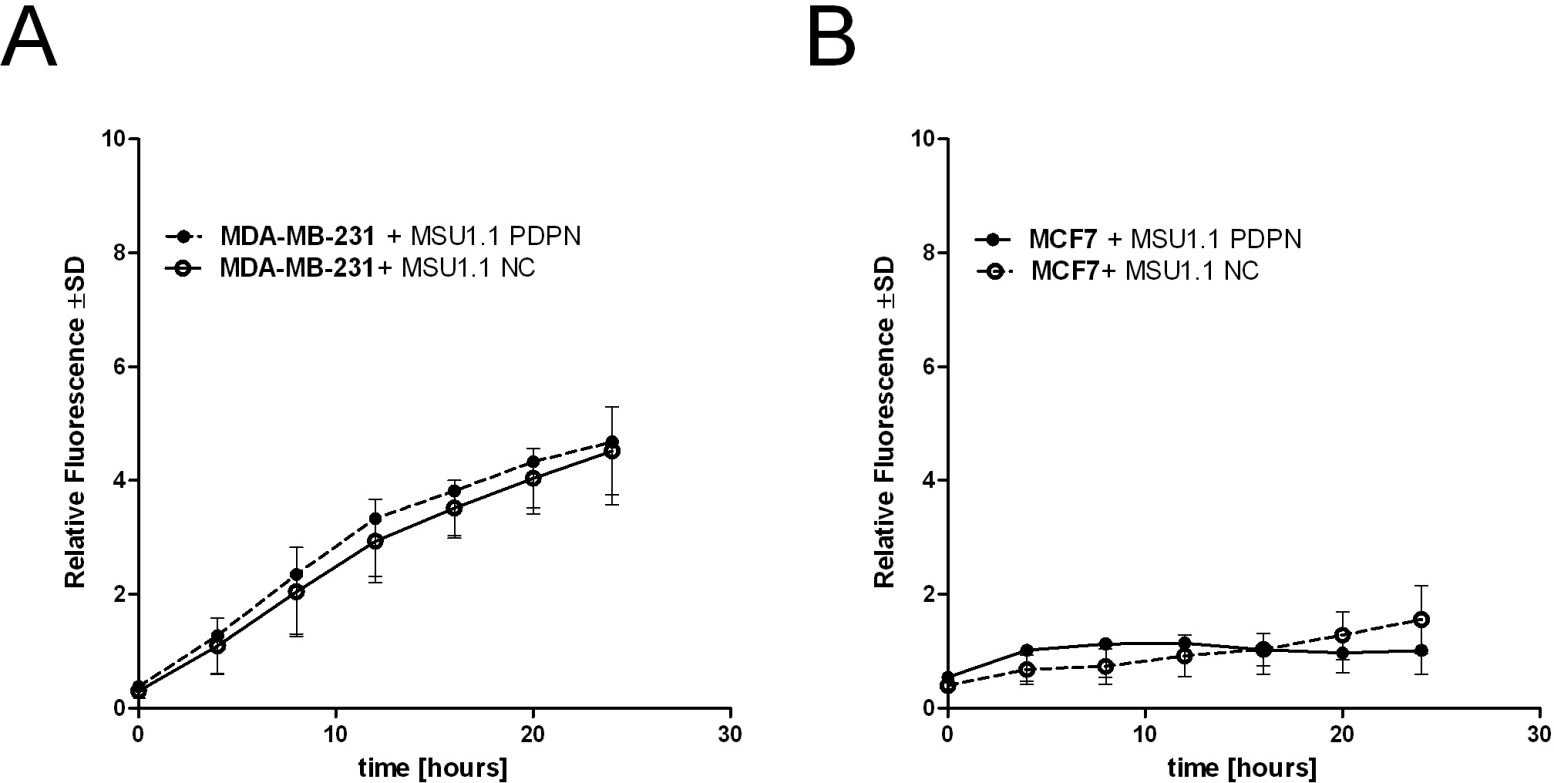
Migratory capability of human breast cancer cell lines co-cultured with human fibroblasts expressing different amounts of podoplanin. **(A)** MSU1.1 PDPN fibroblasts overexpressing podoplanin and podoplanin-negative control MSU1.1 NC cells co-cultured with fluorescently labeled MDA-MB-231 breast cancer cells, **(B)** MSU1.1 PDPN fibroblasts overexpressing podoplanin and podoplanin-negative control MSU1.1 NC cells co-cultured with fluorescently labeled MCF7 breast cancer cells. The migratory properties of breast cancer cells were studied using transwell migration assay. Data are present as mean ±SD.

It has been proposed that podoplanin-expressing CAFs stimulate tumor cell migration and invasiveness, however, the role of this glycoprotein in both processes remains unclear [20,27]. Therefore, to clarify the functional role of podoplanin present on the surface of CAFs in breast cancer progression, migration and invasion assays involving co-cultures of tumor cells and fibroblasts expressing different amounts of podoplanin were performed. When migration of fluorescently labeled MDA-MB-231 and MCF7 breast cancer cells co-cultured with MSU1.1 PDPN fibroblasts overexpressing podoplanin or control MSU1.1 NC fibroblasts was analyzed, no differences in the numbers of migrating tumor cells were found between these co-cultures (**Fig 1A and 1B**). It should be mentioned that the numbers of migrating cancer cells in co-cultures were the same as in cultures which consisted only of tumor cells (data not shown). These results indicate that podoplanin present on the surface of fibroblasts does not change the migratory ability of tumor cells.

**Fig 2:**
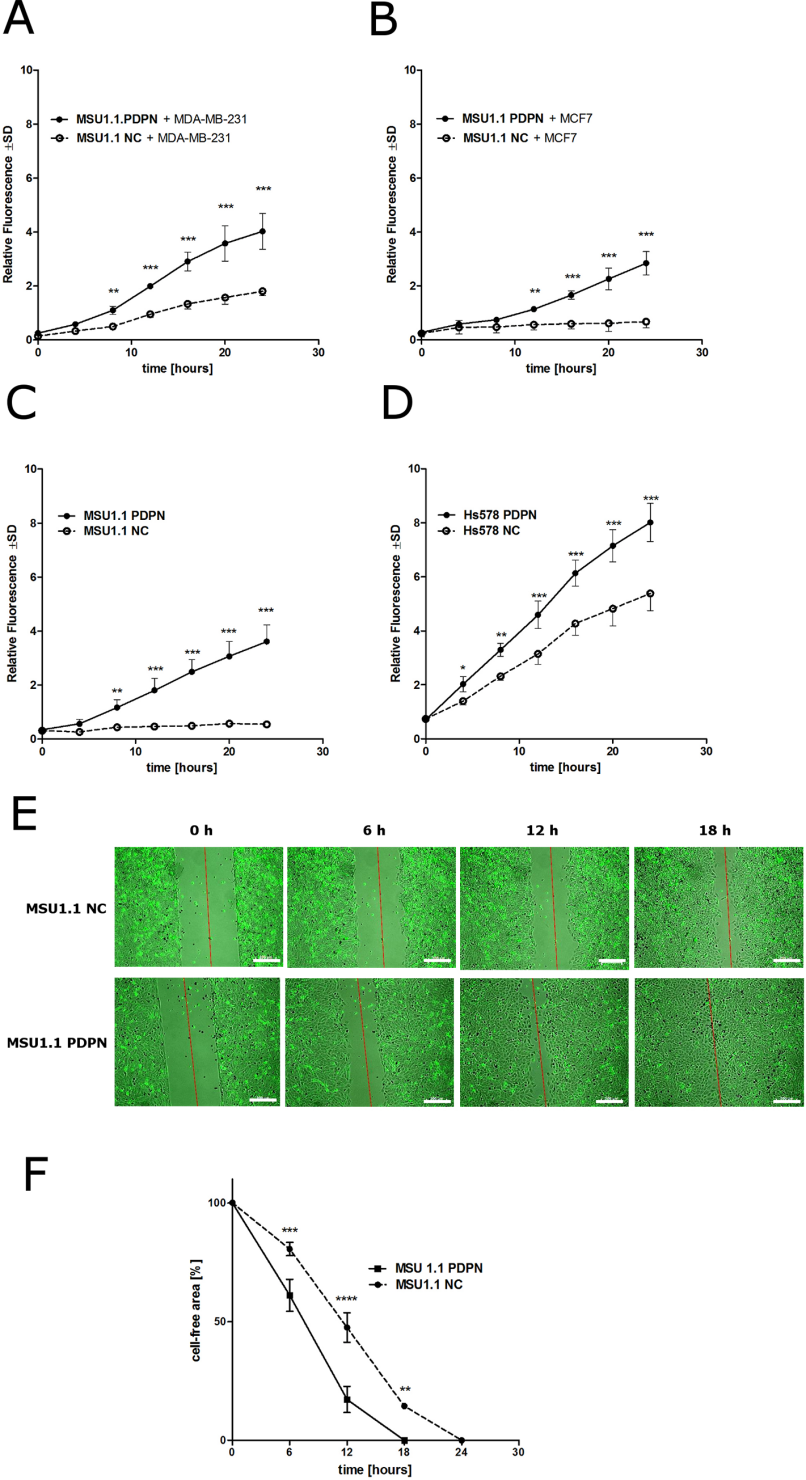
Migratory capability of human fibroblasts expressing different amounts of podoplanin co-cultured with human breast cancer cell lines or cultured alone. Transwell migration assay with fluorescently labeled MSU1.1 PDPN fibroblasts overexpressing podoplanin and podoplanin-negative control MSU1.1 NC fibroblasts co-cultured with MDA-MB-231 breast cancer cells **(A)** or MCF7 breast cancer cells **(B)**; transwell migration assay with MSU1.1 PDPN fibroblasts overexpressing podoplanin and podoplanin-negative control MSU1.1 NC fibroblasts **(C)**, transwell migration assay with Hs578 PDPN fibroblasts overexpressing podoplanin and podoplanin-negative control Hs578 NC fibroblasts **(D)**. Data are presented as mean ±SD. Bonferroni multiple comparison test, *p<0.05, **p<0.01, ***p<0.001, ****p<0.0001. Wound-healing assay with MSU1.1 PDPN fibroblasts overexpressing podoplanin and podoplanin-negative control MSU1.1 NC fibroblasts. Cell–free gap (500 μm ± 50 μm) was generated in the monolayers of fibroblasts, photographs were taken each hour up to 48 hours after the injury, always from the same place (3 pictures per well) **(E)**, and analyzed using ImageJ software (National Institute of Health) **(F)**. Cell-free area was calculated. Data were analyzed using the Bonferroni multiple comparison test, *p<0.05, **p<0.01, ***p<0.001, ****p<0.0001.

In contrast to the above results, when migratory properties of fluorescently labeled MSU1.1 PDPN and MSU1.1 NC fibroblasts co-cultured with MDA-MB-231 or MCF7 cells were analyzed by *in vitro* migration assay a statistically significant increase in the number of migrating cells was found in the case of fibroblasts overexpressing PDPN (**Fig 2A and 2B**). These data raise the possibilities that (i) tumor cells can affect the migratory properties of podoplanin-expressing fibroblasts or (ii) podoplanin expressed by fibroblasts increases their mobility as it has been shown for some other cells [10,35,36]. To address these questions we analyzed the migratory potential of MSU1.1 PDPN fibroblasts and Hs578 PDPN fibroblasts overexpressing podoplanin in comparison to control cells transduced only with empty vector. It was found that the numbers of migrating fibroblasts overexpressing podoplanin were similar to the numbers of fibroblasts overexpressing podoplanin co-cultured with breast cancer cells (**Fig 2C and 2D**). These results revealed that podoplanin present on the surface of fibroblasts profoundly effects their migratory abilities. Essentially the same results were obtained, when MSU1.1 PDPN and MSU1.1 NC fibroblasts were subjected to wound-healing assay (**Fig 2E and 2F)**.

**Fig 3:**
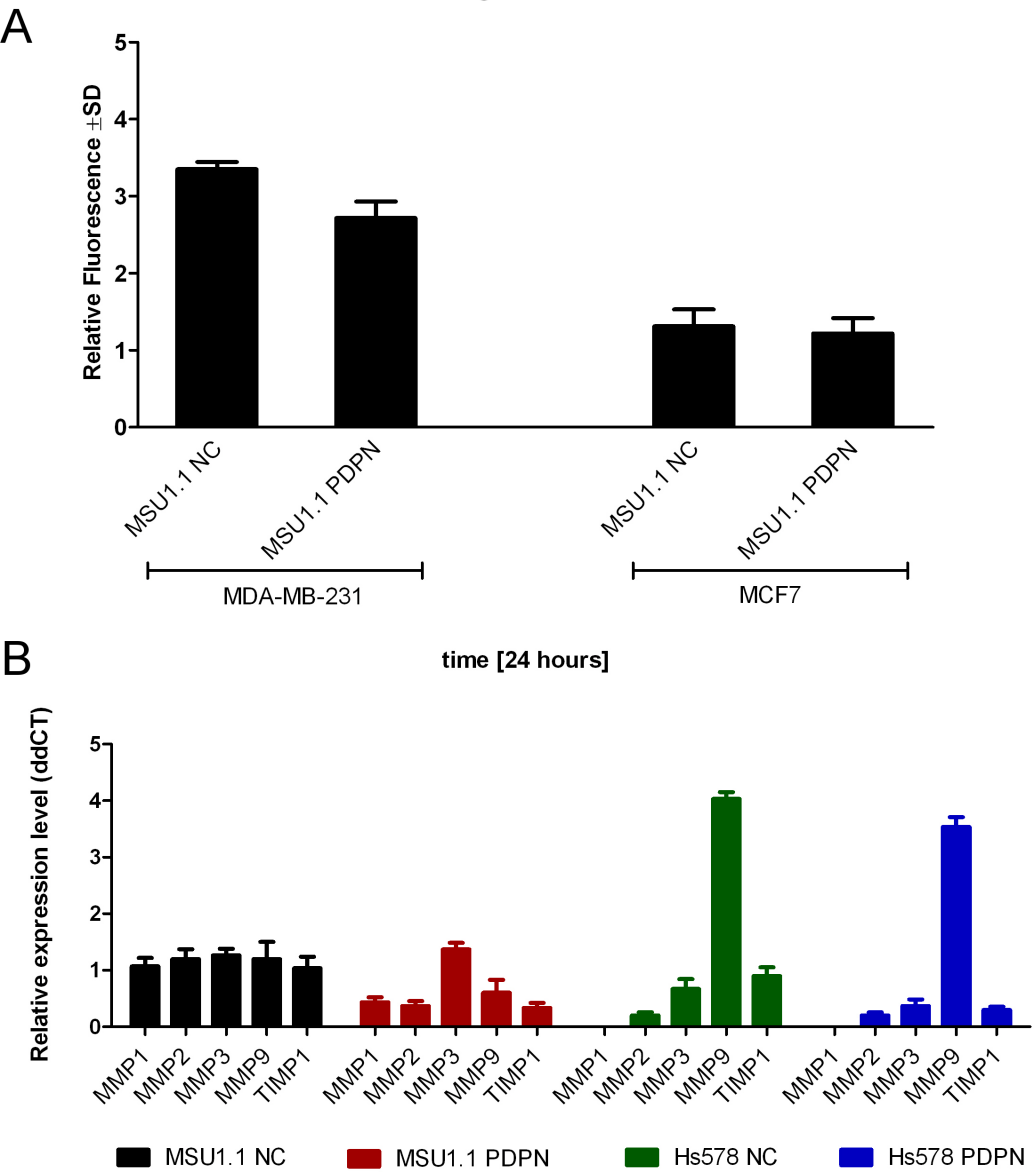
Podoplanin does not affect the invasiveness of breast cancer cell lines. **(A)** Invasiveness of breast cancer cell lines co-cultured with fibroblastic cells expressing different amounts of podoplanin. Invasiveness of fluorescently labeled MDA-MB-231 and MCF7 breast cancers analyzed by Matrigel^TM^ invasion assay in co-cultures with control podoplanin-negative MSU1.1 NC cells and MSU1.1 PDPN cells overexpressing podoplanin. Data are presented as mean ±SD. **(B)** Expression of MMPs and TIMP1 in fibroblastic cells expressing different amounts of podoplanin. Relative expression of MMPs and TIMP1 gene was normalized against expression of *ACTB* gene and MSU1.1 NC cells were assigned as a calibrator sample. Results are expressed as mean ± SD.

Since it was proposed that podoplanin-expressing CAFs not only affect the motility, but also increase the invasiveness of tumor cells [20,27], we analyzed the effect of podoplanin expressed by fibroblastic cells on the invasiveness of breast cancer cells using *in vitro* Matrigel^TM^ invasion assay. No differences in invasiveness were found in the case of fluorescent MDA-MB-231 cells and MCF7 co-cultured with fibroblastic MSU1.1 cells expressing different amounts of podoplanin (**Fig 3A**). These data were supported by the analysis of matrix metalloproteinases (MMPs) expression in MSU1.1 PDPN and Hs578 PDPN fibroblasts overexpressing podoplanin and corresponding control podoplanin-negative cells. Using real-time PCR, no statistically significant differences in the MMP gene expression were found among the analyzed cell populations (**Fig 3B**).

### Podoplanin increases the migratory properties of fibroblasts in ezrin- and RhoA-dependent manner

**Fig 4:**
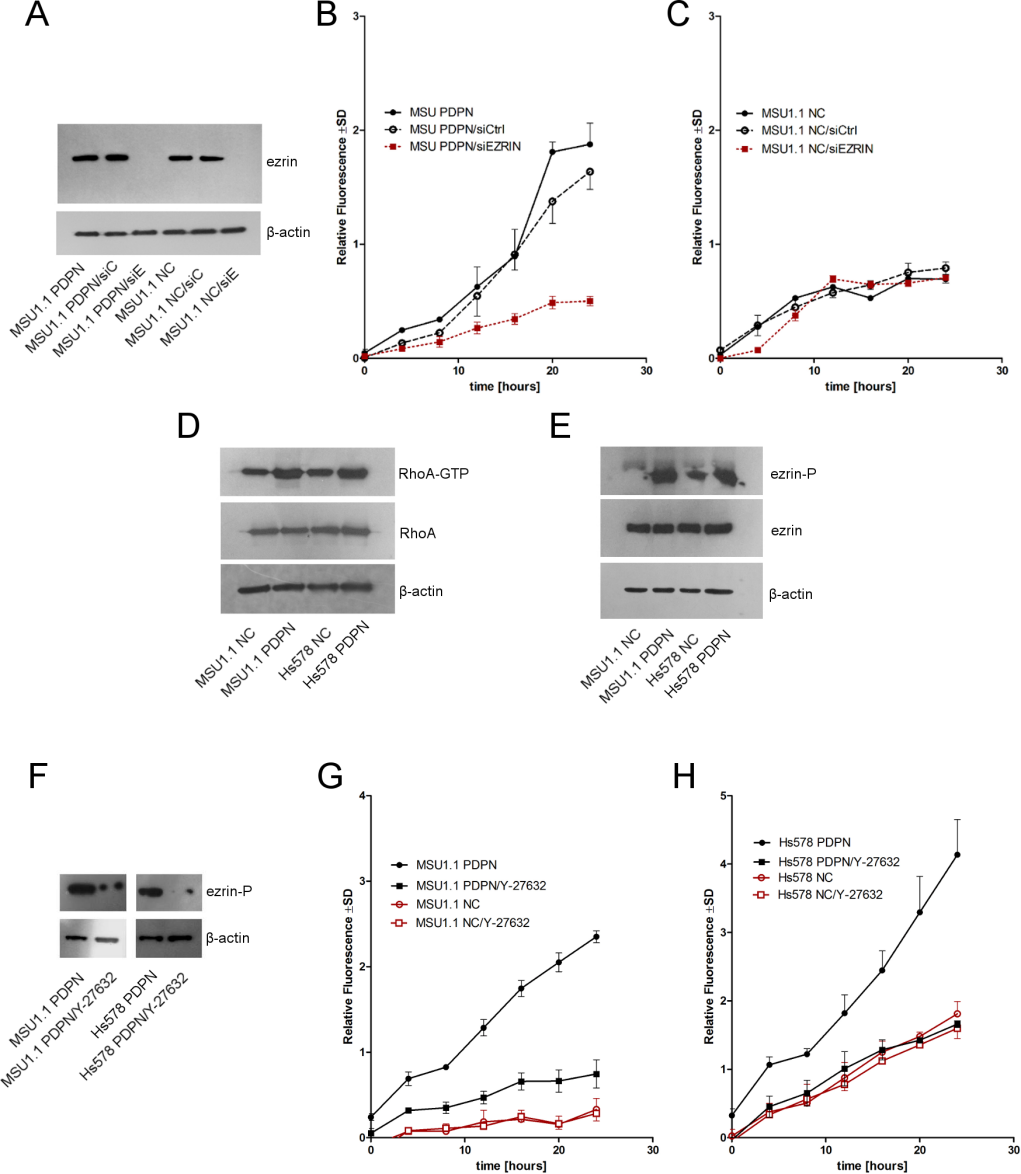
Ezrin and ROCK affects the migratory properties of human MSU1.1 fibroblasts overexpressing podoplanin. **(A)** Expression of ezrin in podoplanin-rich native MSU1.1 fibroblasts (MSU1.1 PDPN), podoplanin-rich MSU1.1 fibroblasts transfected with scrambled siRNA (MSU1.1 PDPN/siC), podoplanin-rich MSU1.1 fibroblasts transfected with siRNA directed against ezrin mRNA (MSU1.1 PDPN/siE), podoplanin-negative native MSU1.1 fibroblasts (MSU1.1 NC), podoplanin-negative MSU1.1 fibroblasts transfected with scrambled siRNA (MSU1.1 NC/siC), and podoplanin-negative MSU1.1 fibroblasts tran/siE, and MSU1.1 PDPN fibroblasts, **(C)** Migratory properties of podoplanin-negative MSU1.1 NC/siC, MSU1.1 NC/siE, and MSU1.1 NC fibroblasts. Transwell migration assay. Data are presented as mean ±SD, **(D)** Expression of ezrin and its phosphorylated form (Thr567) in podoplanin-rich MSU1.1 PDPN and Hs578 PDPN fibroblasts in comparison to controlMSU NC and Hs578 NC cells. Ezrin and phosphorylated ezrin (pThr5567) were detected by Western blotting using specific rabbit polyclonal antibodies. β-Actin served as an internal control. (**E**) Western blotting analysis of active RhoA-GTPases relative to the total expression levels of RhoA in podoplanin-rich MSU1.1 PDPN and Hs578 PDPN fibroblasts in comparison to control MSU1.1 NC and Hs578 NC cells. The levels of β-actin were determined as a control for protein loading. (**F**) Expression of phosphorylated ezrin (Thr567) in podoplanin-rich MSU1.1 PDPN and Hs578 PDPN fibroblasts treated with specific Y-27632 inhibitor of ROCK. Phosphorylated ezrin (pThr5567) was detected by Western blotting using specific rabbit polyclonal antibodies. (**G**) Migratory properties of podoplanin-rich MSU1.1 PDPN fibroblasts grown in the presence of Y-27632 inhibitor. **(H)** Migratory properties of podoplanin-rich Hs578 PDPN fibroblasts grown in the presence of Y-27632 inhibitor. Transwell migration assay. Data are presented as mean ±SD.

It has been proposed that podoplanin affects the migratory properties of cells in ezrin- and RhoA-dependent manner [11]. Therefore, to show that podoplanin-dependent migratory properties of fibroblastic cell lines are dependent directly on its interaction with ezrin, we created a specific loss-of-function phenotype using siRNAs to inhibit the expression of ezrin in MSU1.1 fibroblasts with high expression of podoplanin (MSU1.1 PDPN) and control MSU1.1 cells (MSU1.1 NC). The cells transfected with specific anti-ezrin siRNAs were characterized by highly decreased levels of ezrin mRNA and highly decreased binding of anti-ezrin antibodies to cell lysates in comparison to control cells transfected with scrambled siRNA (**Fig 4A**). When such fibroblastic cell lines were subjected to transwell migratory assay, highly diminished migratory capabilities of podoplanin-rich cells with low expression of ezrin in comparison to podoplanin-expressing cells with unchanged expression of ezrin were observed (**Fig 4B and 4C**). In the next step, we analyzed the activity of RhoA in fibroblasts with high and no-expression of PDPN, and found that the activity of RhoA and the level of ezrin phosphorylation were higher in podoplanin-rich fibroblasts (**Fig 4D and 4E**). In addition, as upregulation of RhoA activity leads to ROCK (RhoA-associated kinase)-mediated ERM phosphorylation, the activity of ROCK was inhibited with specific Y-27632 inhibitor. As anticipated, phosphorylation of ezrin was highly diminished (**Fig 4F**) and migration of PDPN-expressing fibroblasts was blocked (**Fig 4G and 4H**).

### Podoplanin affects the cellular distribution of ezrin and Rho-GDI in fibroblastic cells

**Fig 5:**
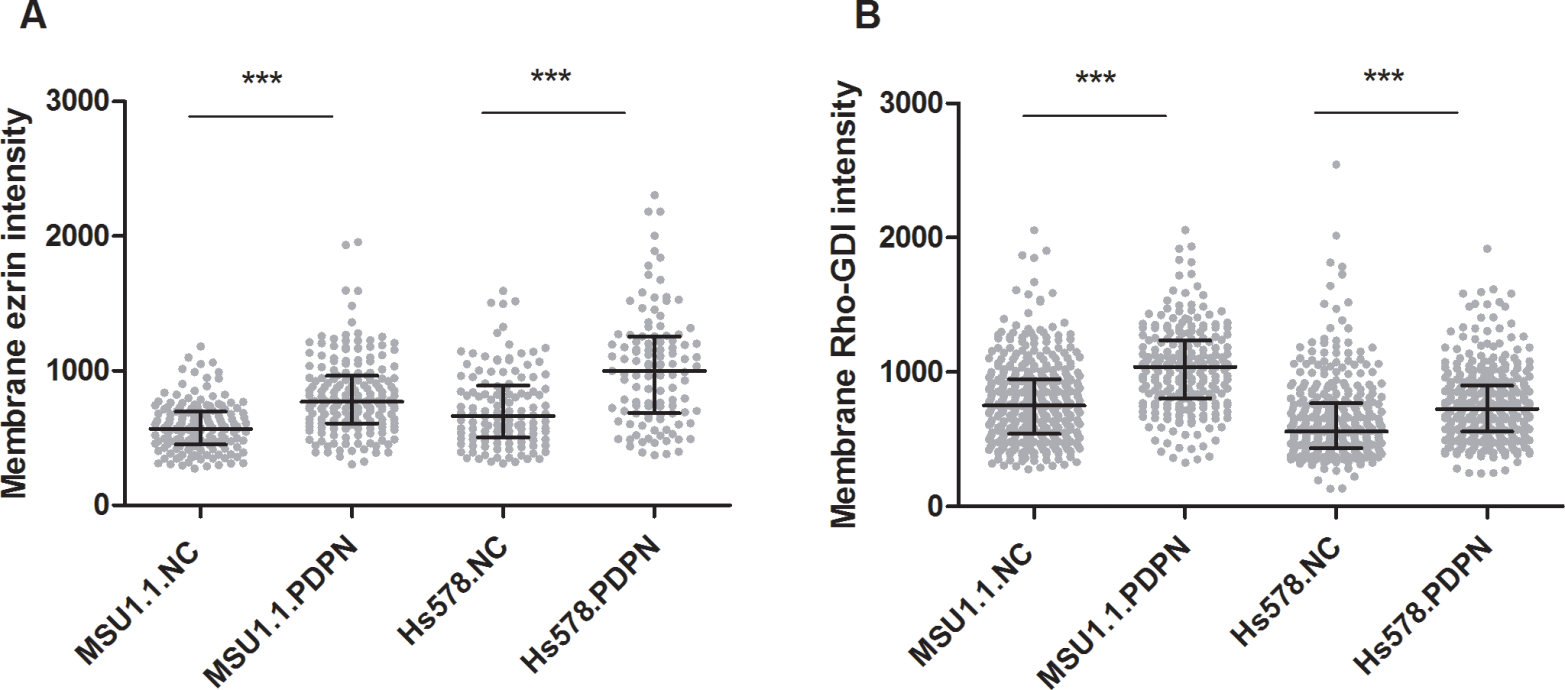
Immunofluorescent analysis of podoplanin-regulated ezrin and Rho-GDI presence in plasma membrane of MSU1.1 and HS578T cells. Podoplanin-negative and podoplanin-overexpressing MSU1.1 and HS578T cells were immunofluorescently labelled to visualize distribution of ezrin and Rho-GDI proteins. Based on plasma membrane and cytoplasm co-staining with WGA-Alexa Fluor 647 and CTV, respectively, ezrin **(A)** and Rho-GDI **(B)** content in plasma membrane was quantified with confocal microscopy and image analysis using ImageJ software. Data are presented as mean fluorescence intensities in identified plasma membrane regions (single dots). Data were analyzed using the Mann–Whitney test, *p<0.05, **p<0.01, ***p<0.001, ****p<0.0001.

It was proposed that activation of RhoA protein by podoplanin first involves formation of podoplanin-ezrin protein complex at the cell membrane, which then recruits Rho-GDI associated with RhoA-GDP. Such interaction causes the release of RhoA-GTP, what in turn allows for RhoA activation by the GDP exchange for GTP [11]. To investigate effects of podoplanin on cellular distribution of ezrin and Rho-GDI we resorted to confocal microscopy. To this end we used podoplanin-negative and podoplanin-overexpressing MSU1.1 and Hs578Bst cell cultures immunofluorescently labelled with specific antibodies directed against ezrin and Rho-GDI. First, we analyzed ezrin location in cells co-stained with WGA-Alexa Fluor 647 conjugate, showing plasma membrane, and CTV intravital dye, which accumulates in cytoplasm. The image analysis was performed on equatorial optical sections providing distinct outlines of plasma membrane. **Fig 5A** shows quantification of ezrin fluorescence intensity in identified plasma membrane regions. We found that fibroblastic cells overexpressing podoplanin have consistently increased level of ezrin, compared to podoplanin-negative cells. Then we analyzed in a similar way, cellular distribution of Rho-GDI protein and found that fibroblasts expressing podoplanin also consistently accumulate this protein in plasma membrane only in the presence of podoplanin (**Fig 5B**).

### Podoplanin present on the surface of fibroblasts affects the pseudo tube formation by HSkMEC endothelial cells

**Fig 6:**
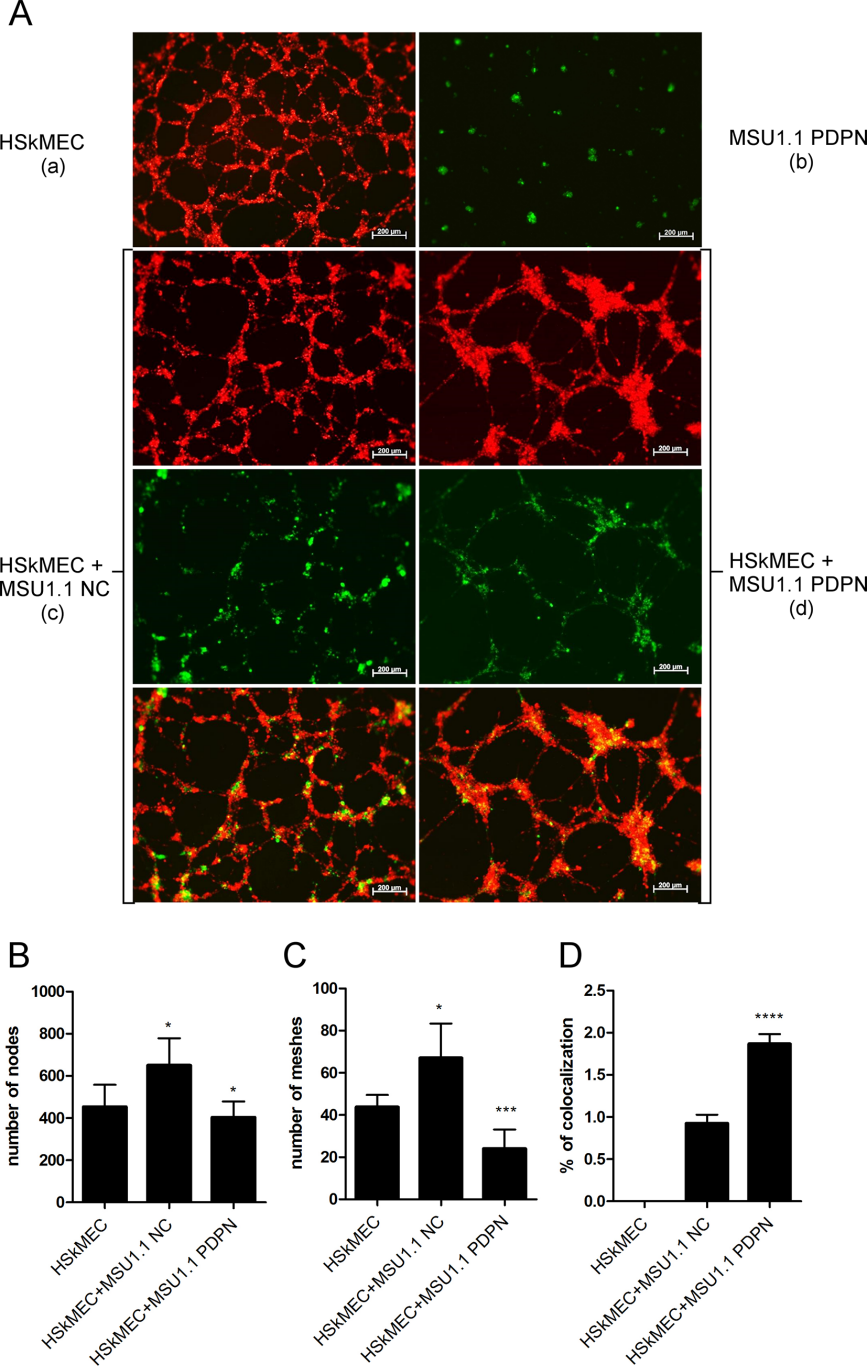
Effect of podoplanin expressed in fibroblastic MSU1.1 cells on the pseudo tub formation properties of endothelial HSkMEC cells in co-culture. **(A)** Pseudo tube formation Matrigel^TM^ assay of **(a)** fluorescently labeled (DiD) endothelial HSkMEC cells, **(b)**fluorescently labeled (DiO) MSU1.1 PDPN fibroblasts overexpressing podoplanin,**(c)** fluorescently labeled (DiD) endothelial HSkMEC cells co-cultured with fluorescently labeled (DiO) control podoplanin-negative MSU1.1 NC fibroblasts and **(d)** fluorescently labeled (DiD) endothelial HSkMEC cells co-cultured with fluorescently labeled (DiO) MSU1.1 PDPN fibroblasts overexpressing podoplanin. Images were obtained in the red channel for DiD labelling of HSkMEC cells, in the green channel for the DiO labelling of MSU1.1 NC and MSU1.1 PDPN and the merge for the co-localization. Images were analyzed after 12 h of assay duration. **(B)** Number of nodes and **(C)** number of meshes formed by HSkMEC endothelial cells co-cultured with MSU1.1 PDPN fibroblasts overexpressing podoplanin and podoplanin-negative control MSU1.1 NC fibroblasts. **(D)** Co-localization of MSU1.1 PDPN and MSU1.1 NC fibroblastic cells with endothelial HSkMEC cells. The number of nodes, meshes and percentage of fibroblasts co-localized with endothelial cells were estimated using ImageJ software.

Podoplanin expressed on CAFs affects the biological properties of tumor cells [20,27]. However nothing is known about its effects on other cells present in tumor stroma, including endothelial cells. To approach this problem, we performed the Matrigel^TM^ pseudo tube formation assay with human skin microvascular endothelial HSkMEC cells co-cultured with human MSU1.1 fibroblasts overexpressing podoplanin. The co-cultures of endothelial cells (EC) with podoplanin expressing MSU1.1 PDPN fibroblasts presented much more disordered capillary-like network characteristic for cancer vasculature [37] than co-cultures of EC with control MSU1.1 NC fibroblasts (**Fig 6A**). These observations are reflected in the numbers of nodes/branches and meshes/loops as a measure of *in vitro* endothelial activity to form network. When HSkMEC cells were co-cultured with podoplanin-rich MSU1.1 PDPN fibroblasts the EC capillary-like network was characterized by significantly lower numbers of nodes and meshes than in co-cultures of HSkMEC endothelial cells with podoplanin-negative MSU1.1 NC fibroblasts (**Fig 6B and 6C**).

The Matrigel^TM^ pseudo tube formation assay was also used to study the co-localization of MSU1.1 PDPN and MSU1.1 NC fibroblasts co-cultured with endothelial cells. It was found that the percentage of MSU1.1 fibroblasts overexpressing podoplanin, which co-localized with HSkMEC cells was twice higher than the number of control MSU1.1 cells which do not express PDPN (**Fig 6D**).

### Expression of angiogenic factor genes in fibroblastic cells is affected by the presence of PDPN

**Fig 7:**
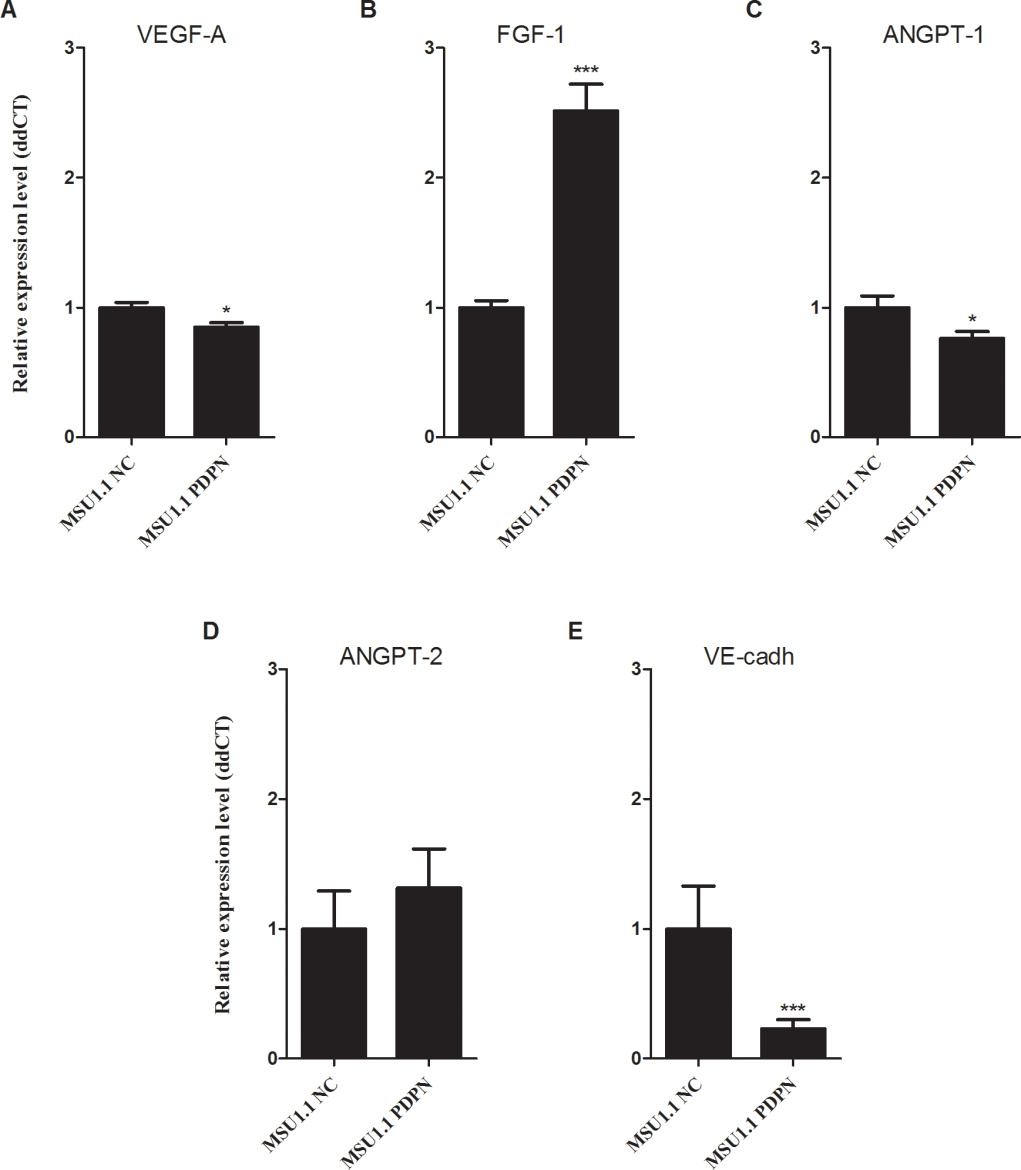
Expression of proangiogenic factors mRNA in fibroblastic cell lines: MSU1.1 PDPN overexpressing podoplanin and control MSU1.1 NC transduced with vector alone. **(A)** VEGF-A, **(B)** FGF-1, **(C)** ANGPT-1, **(D)** ANGPT-2, **(E)** VE-cadherin. Real-time RQ-PCR was used to analyze proangiogenic factors mRNA. Proangiogenic factors levels were normalized against *ACTB* gene expression and cell line MSU1.1 NC was assigned as a calibrator sample. Each bar represents mean ± SEM, * *p* < 0.05, ** *p* < 0.01, **** *p*< 0.0001.

To support the finding that PDPN expressed by fibroblasts affects the pseudo tube formation, we analyzed the expression of angiogenic factor genes produced by fibroblasts overexpressing podoplanin in comparison to control PDPN-negative fibroblasts. Using real-time PCR, it was found that the expression of PDPN in MSU1.1 PDPN cells increased the level of FGF-1 and Angpt2 mRNAs (however, in case of Angpt2 the difference was not statistically significant), and decreased the level of Angpt1 and VE-cadherin gene expression, not affecting the expression of VEGF-A mRNA (**Fig 7**).

### Expression of podoplanin in DCIS and IDC samples

**Fig 8:**
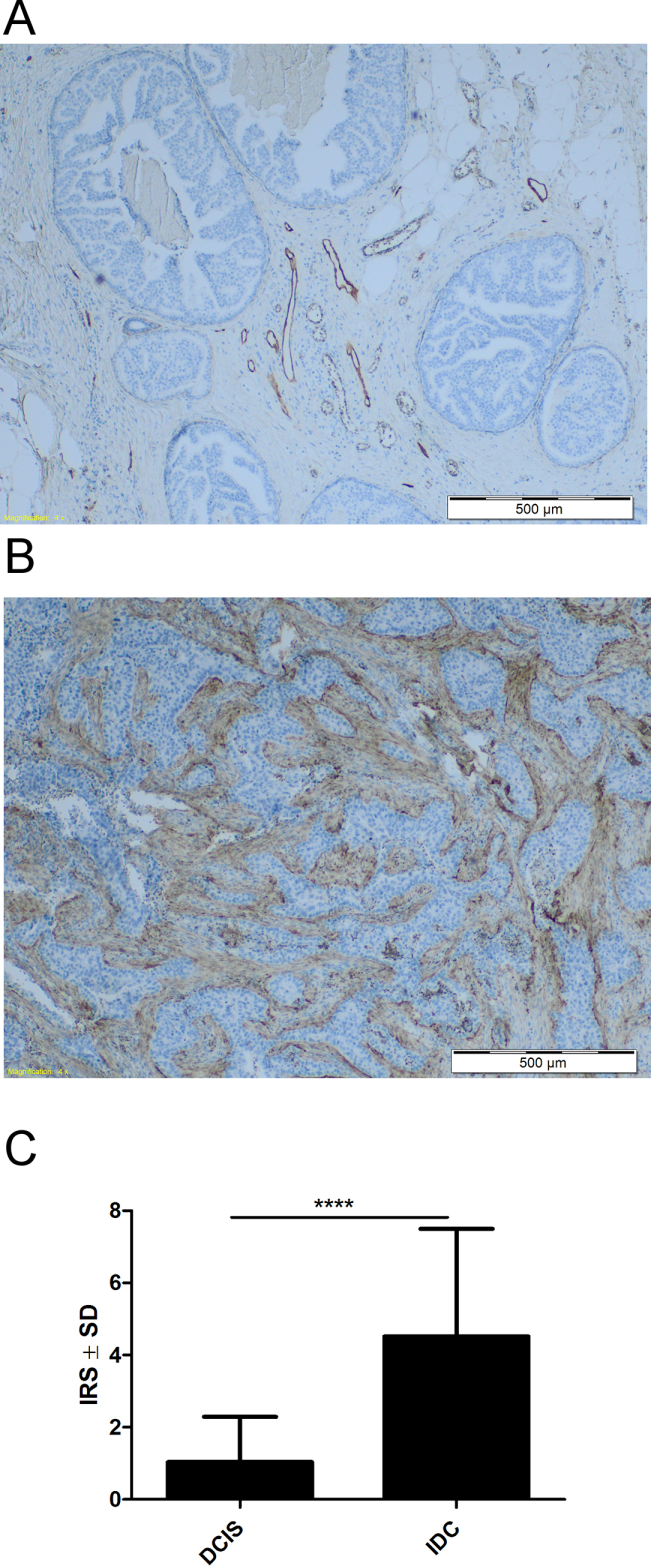
Immunohistochemical staining of podoplanin expressing CAFs in DCIS and IDC. **(A)** The lack of podoplanin expression (D2-40) in CAFs of ductal carcinoma in situ (DCIS). In the center numerous lymphatic vessels D2-40+ are seen. **(B)** Strong podoplanin expression in stromal CAFs of invasive ductal carcinoma (IDC). **(C)** The intensities of podoplanin expression in stromal CAFs of DCIS (*n* = 27) and IDC (*n* = 95).****p<0.0001 for podoplanin-expressing IDC as compared with DCIS (Mann-Whitney *U*-test). Reaction intensities with murine monoclonal anti-human D2-40 antibody were calculated on the basis of the semi-quantitative IRS scale of Remmele and Stegner [33] and are presented as means.

**Fig 9:**
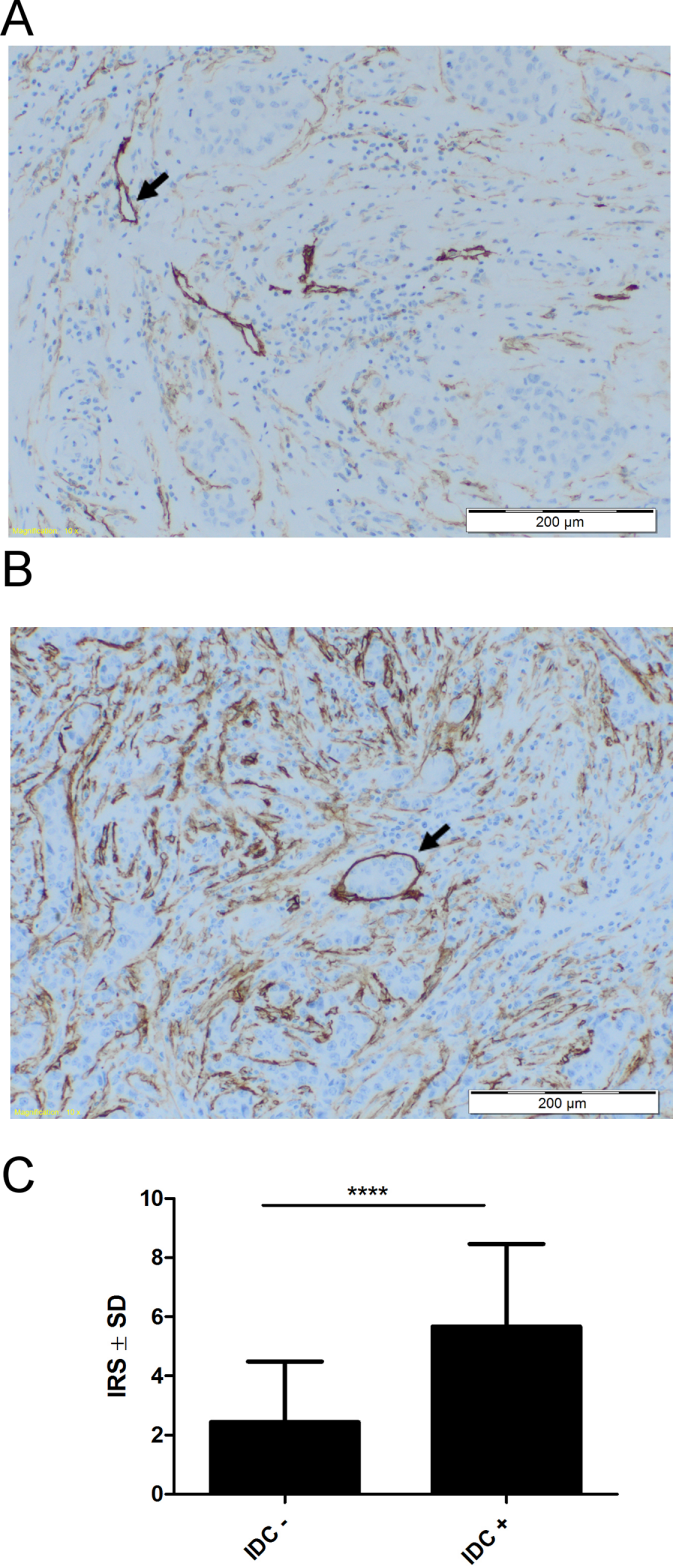
Immunohistochemical staining of podoplanin expressing CAFs in invasive ductal carcinoma without lymphatic vessel invasion by cancer cells (IDC-) and invasive ductal carcinoma with lymphatic vessel invasion by cancer cells (IDC+). **(A)** Weak expression of podoplanin (D2-40) in stromal CAFs of (IDC-). Lymphatic vessels D2-40+ (arrow). **(B)** Strong podoplanin expression in stromal CAFs of (IDC+). Lymphatic vessel with cancer cells (arrow). **(C)** The intensities of podoplanin expression in stromal CAFs of IDC- (n = 34) and IDC+ (n = 61). ****p<0.0001 for strong podoplanin-expressing IDC+ as compared with weak podoplanin-expressing IDC- (Mann-Whitney *U*-test). Reaction intensities with murine monoclonal anti-human D2-40 antibody were calculated on the basis of the semi-quantitative IRS scale of Remmele and Stegner [33] and are presented as means.

The expression of podoplanin in CAFs was studied in DCIS and IDC cases. It was found that expression of this glycoprotein was significantly higher in IDC than in DCIS cases (*p* < 0.0001) (**Fig 8**). Furthermore, when IDC cases were divided into two groups: with lymphatic vessel invasion by cancer cells (IDC+) and cases without invasion (IDC-), the podoplanin expression in CAFs was significantly higher in IDC+ than in IDC- cases (*p* < 0.0001) (**Fig 9).**

### Podoplanin-positive CAFs in IDC peripheral and tumor stroma

**Fig 10:**
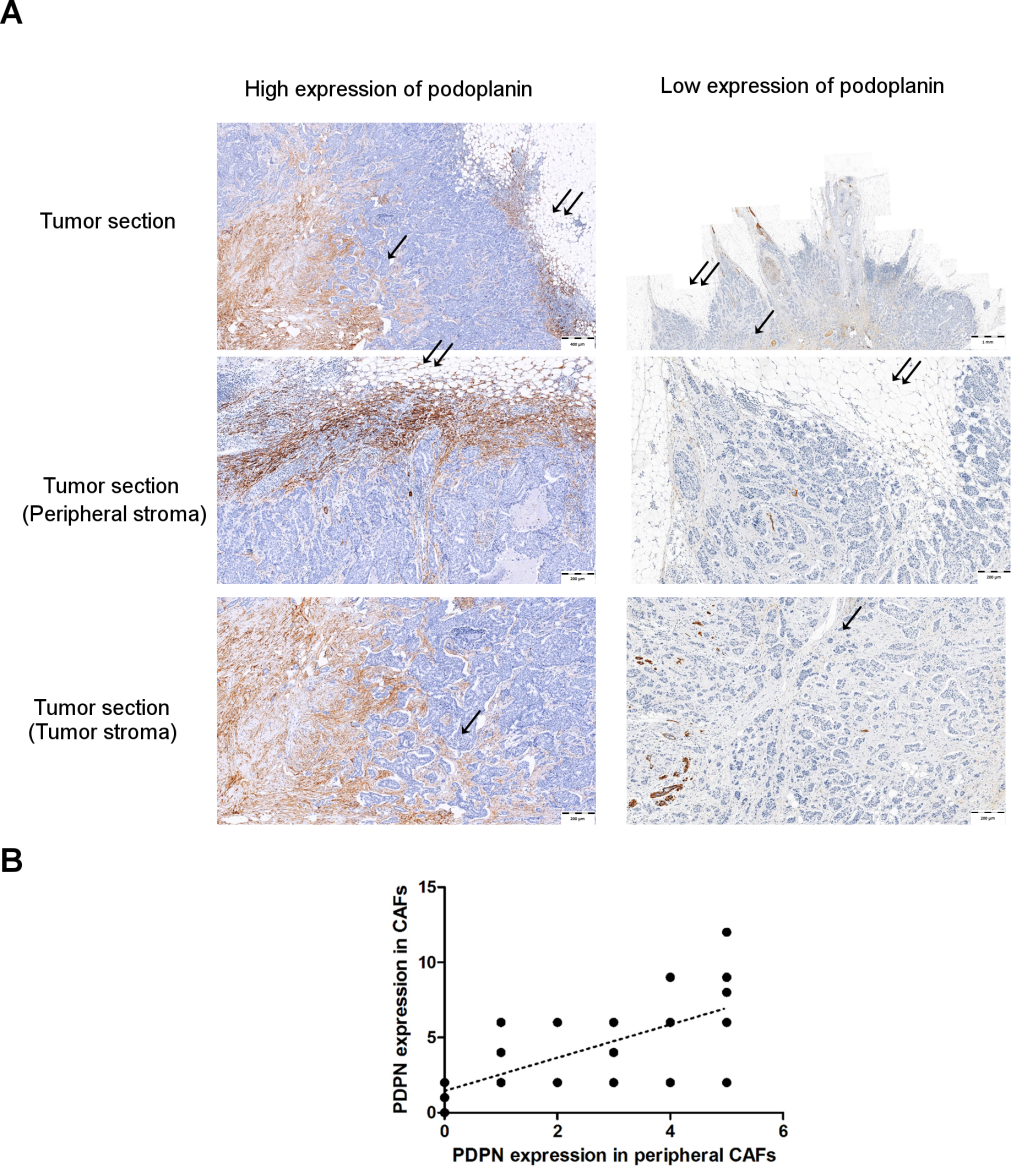
Immunohistochemical staining of podoplanin-positive CAFs in IDC peripheral and tumor stroma. **(A)** IDC with high and low expression level of podoplanin (D2-40). **(B)** Positive correlation between podoplanin (D2-40) expression in tumor stroma and podoplanin (D2-40) present in peripheral stroma at the margin of tumor. r = 0.694, *p* < 0.0001 (Spearman’s correlation test). For the evaluation of podoplanin expression intensity the semiquantitative immunoreactive score (IRS) of Remmele and Stegner [33] was used.

Using IHC, we also analyzed the presence of podoplanin expressing CAFs in peripheral stroma at the margin of tumor and tumor stroma in the same paraffin sections of invasive ductal carcinoma (IDC). In many (12/29) IDC cases, high expression of podoplanin in peripheral stroma was associated with high expression of podoplanin in the tumor stroma. On the other hand, when the podoplanin expression was low in peripheral stroma, there was also low podoplanin expression in tumor stroma (**Fig 10A**). Therefore, significant positive correlation (r = 0.694, *p* < 0.0001) between the expression of podoplanin in peripheral stroma at the margin of tumor and tumor stroma was found using Spearman’s correlation analysis (**Fig 10B**).

### PDPN-positive CAFs co-localize with blood vessels in IDC tissue specimens

**Fig 11:**
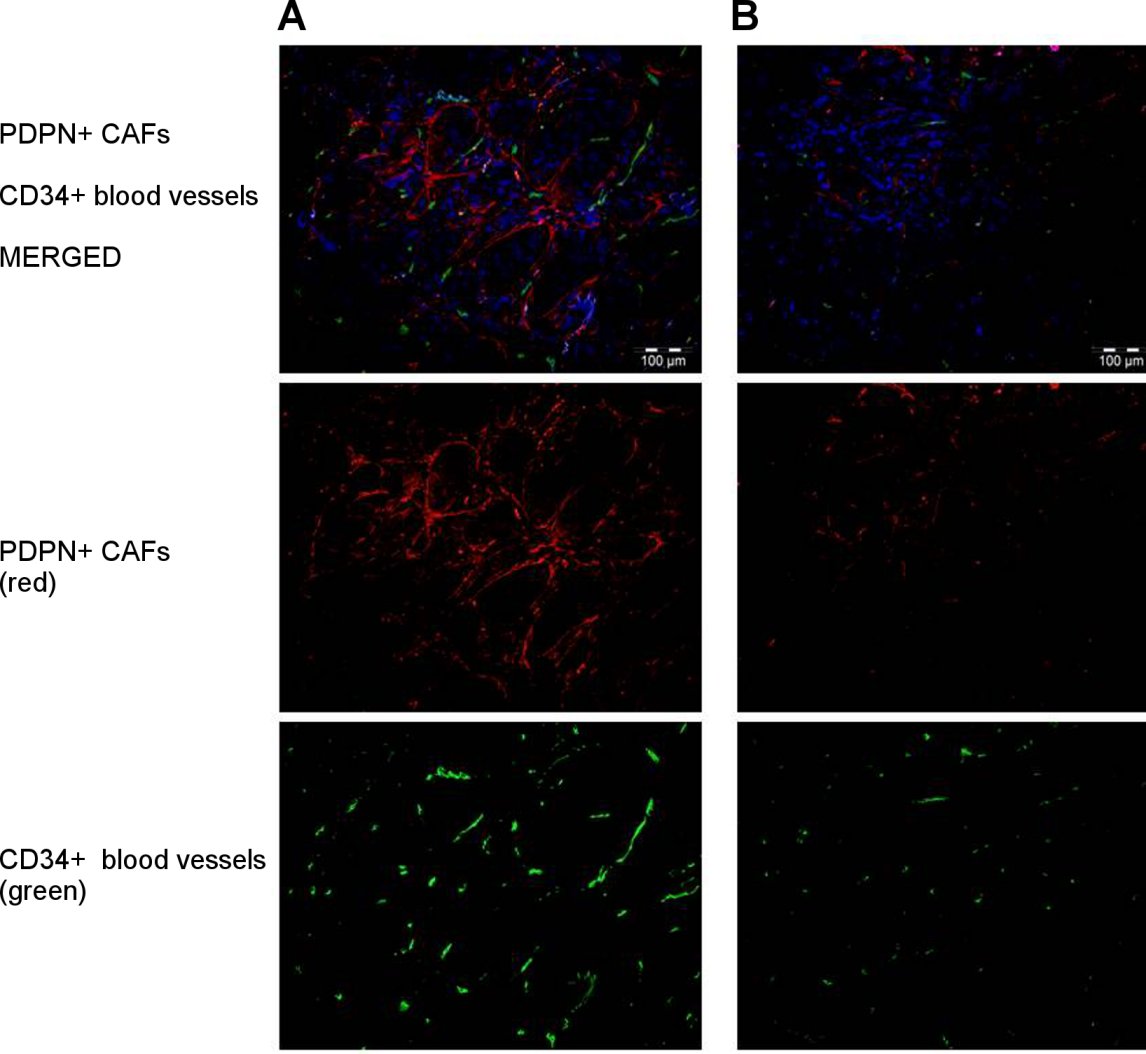
Immunofluorescent co-localization of PDPN-positive CAFs and CD34-positive blood vessels in IDC stroma. **(A)** IDC with high expression of podoplanin, **(B)** IDC with low expression of podoplanin. Anti-podoplanin antibody was stained with secondary donkey anti-mouse antibodies conjugated with Alexa-Fluor 594 (red), and anti-CD34 antibody was detected with donkey anti-rabbit Alexa-Fluor 488 conjugated secondary antibody (green).

Using IF, we analyzed the co-localization of PDPN-positive CAFs with blood vessels stained with antibody directed against CD34 in tumor stroma of IDC samples. High level of podoplanin expression was observed in the vicinity of numerous blood vessels present in tumor stroma, and weak staining with antibody directed against PDPN was found in the proximity of single blood vessels (**Fig 11**).

## Discussion

Recent studies on ductal breast cancer revealed that the presence of CAFs expressing podoplanin positively correlated with tumor size, degree of malignancy, lymph node metastases, invasion into lymphatic and blood vessels, expression of Ki67 antigen, shorter patients’ survival and VEGF-C expression [14,18,38]. Based on these results, the present study was undertaken to assess on the molecular level if and how podoplanin expressed by fibroblasts can affect malignancy-associated properties of breast cancer cells and to understand the role of podoplanin expressed by fibroblasts/CAFs in breast cancer progression.

CAFs present in tumor stroma may promote cancer progression by secretion of numerous chemokines, growth factors, enzymes and other proteins, which among other activities induce angiogenesis, recruit progenitor endothelial cells from bone marrow, or participate in extracellular matrix remodeling [23,25]. Therefore in cancer cell biology it is very difficult to define the specific role of individual proteins expressed by CAFs, including podoplanin, on properties such as proliferation, survival, invasion and ability to metastasize. For example, using the transwell co-culture system [39], it was found that migratory and invasive properties of pancreatic cancer cells were much higher when they were co-cultured with stromal fibroblasts highly expressing podoplanin. However, an inhibitory study revealed that the absence of podoplanin on stromal fibroblasts has no effect on migratory and invasive properties of pancreatic cancer cells, indicating the lack of a functional link between podoplanin expression and their biological properties. This suggests that podoplanin is only a marker of a specific subpopulation of fibroblasts, which interacts with cancer cells. Therefore, to avoid the influence of other factors/proteins produced by CAFs and to determine if the biological behavior of breast cancer cells can be affected only by the presence of podoplanin, we used previously established fibroblastic cell lines. MSU1.1 cells are normal, non-activated skin fibroblasts, characterized by the expression of vimentin and FSP protein, and low expression of α-SMA. Hs578Bst fibroblasts, which were derived from normal breast tissue surrounding ductal carcinoma, contain microfilaments and clusters of pinocytotic vesicles suggesting their myoepithelial origin. However, their antigenic profile was similar to MSU1.1 cells. The study was performed by constructing “gain-of-function” phenotypes since neither cell line expresses podoplanin. Our assumption was that, if cells with ectopic expression of podoplanin have different properties in comparison to parental cells then these differences can be directly linked to the presence of this glycoprotein.

So far, the effects of podoplanin present on the surface of CAFs on the biological properties of cancer cells have been studied in the case of human lung adenocarcinoma [21,26,27] and invasive ductal carcinoma of the pancreas [20]. Using nude mice it was found that human vascular adventitial fibroblasts (hVAFs) expressing high level of podoplanin increase the ability of human lung adenocarcinoma cells to develop tumors and form lung metastases as compared to human lung fibroblasts (hLFs) with low expression of this glycoprotein [21]. Using a newly developed collagen invasion assay, Neri et al. [27] found that CAFs highly expressing podoplanin were actively invasive, and cancer cell movement corresponded to the “tracks” created by these CAFs. It was also shown that PDPN present on the surface of fibroblasts increases the motility and viability of melanoma cells [40]. In contrast to the above studies, we found using the transwell co-culture system that migratory and invasive properties of two breast cancers MDA-MB-231 and MCF7 cell lines, which are characterized, respectively, as highly migratory and non-migratory, were not affected by the presence of podoplanin on the surface of fibroblasts. However, we showed that ectopic expression of podoplanin highly increases the migration of MSU1.1 fibroblasts, which are only weakly migratory and increases the migration of highly-migratory Hs578Bst fibroblasts. As clinical data showed the association between the presence of podoplanin-positive CAFs and the progression of breast cancer [14], we propose that expression of podoplanin by fibroblasts facilitates their movement into the tumor stroma, what increases the number of CAFs and creates a favorable microenvironment for tumor progression by facilitating local invasion of cancer cells. This hypothesis was supported by clinical studies. First, the PDPN-positive fibroblasts were found in peripheral stroma at the margin of tumor and their numbers correlated with numbers of podoplanin expressing CAFs in tumor stroma, what suggests that at least part of PDPN-positive CAFs comes from outside the tumor. However, we cannot exclude the possibility that actually, tumor-born PDPN-positive fibroblasts with high motility migrate efficiently from tumor to surrounding tissues, helping cancer cells during their invasion. Second, when invasive ductal carcinomas (IDC) and non-invasive ductal carcinomas in situ (DCIS) were analyzed by immunohistochemistry for the presence of podoplanin-expressing CAFs, the numbers of CAFs with high expression of this glycoprotein were significantly higher in IDC cases than in DCIS cases. Furthermore, when IDC cases were divided into two groups: with lymphatic invasion (IDC+) and with no-lymphatic invasion (IDC-), the intensity of CAFs staining by anti-podoplanin antibody was much stronger in case IDC+. Similar clinical data were obtained by Shindo et al. [20] in the case of invasive ductal carcinoma of the pancreas, as well as Neri et al. [27] in the case of lung adenocarcinoma. However, migratory properties of podoplanin-rich *vs*. podoplanin-poor fibroblasts were not analyzed by these authors, and Neri et al. [27] did not find differences in the MMP activity in CAFs with different expression of podoplanin. We thus propose that podoplanin increases the invasive properties of fibroblasts by increasing their migratory properties without affecting the expression of matrix metalloproteinases, which allow them to “colonize” tumor stroma in higher numbers. This facilitates the creation of an appropriate microenvironment by production of numerous factors affecting proliferation, survival and invasion of cancer cells as well as tumor angiogenesis [12,23–25] or as it was suggested by Neri et al. by physical remodeling of ECM by CAFs [27]. In accordance with this, the present study revealed that such podoplanin-mediated effects can influence the pathological state of the angiogenesis in the tumor site. Podoplanin is described as a specific marker of lymphatic capillaries absent from the normal endothelial cells in blood vessels [41]. It was found that this glycoprotein plays a critical role in correct lymphatic vasculature formation and function [42,43]. It also plays a central role in the separation of blood and lymphatic vasculature [44,45]. So far, there was no indication that podoplanin can affect the angiogenesis of blood vessels. Therefore, we have shown for the first time that podoplanin-rich fibroblasts interact more efficiently with endothelial cells than control fibroblasts with no expression of this glycoprotein. Analysis of tube formation by HSkMEC endothelial cells, which were co-cultured with podoplanin-expressing fibroblasts, suggests that podoplanin influences the normal vascularization and can participate in the creation of an abnormal vascular network typical for tumor stroma in which early endothelial progenitor cells participate [46]. Thus, endothelial cells in the tumor blood vessels might be dedifferentiated enough to express molecules typical for precursors not yet engaged into lymphatic/blood vessels separation. Such an effect may also mean a role for PDPN expression in EndoMT process [47]. This data was further supported by the analysis of angiogenic factor gene expression in fibroblasts upon PDPN expression. It was found that PDPN-rich fibroblasts express more angiopoietin 2, while angiopoietin 1 is down regulated. As these molecules compete for their common receptor Tie2 [48] and Angpt1 is responsible for the attraction of pericytes by endothelial cells forming vessels [49] such changes in expression profile of angiogenic factors corresponds to a characteristic feature of pathologic angiogenesis. Important evidence that PDPN-positive fibroblasts can affect the formation of blood vessels comes from the clinical studies as the presence of large numbers of PDPN-positive CAFs was associated with numerous CD34-positive blood vessels in tumor stroma of IDC samples. On the other hand, in the areas of low PDPN-staining mostly single CD34-positive blood vessels were observed.

Key role of phosphorylated ezrin [8,10,11] in podoplanin-mediated motility of MSU1.1 and Hs578Bst fibroblasts was confirm as siRNA-directed inhibition of ezrin expression blocked the migration of these cells, and substantial amounts of phosphorylated ezrin were present only in migratory podoplanin-expressing fibroblasts. We have also shown that phophorylation of ezrin is RhoA-dependent, as inhibition of RhoA-associated kinase (ROCK) blocked the migration of these cells, what correlated with highly decreased level of phosphorylated ezrin. Ezrin, like other ERM proteins, has the potential to act not only as effectors of RhoGTPases but also as regulators of their activity [50] Their activation may result, among others, from the association of ERM proteins with Rho GDP-dissociation inhibitor (Rho-GDI) [51]. Therefore, in order to explain, how podoplanin activates RhoA protein, a mechanism similar to the one described for CD44 antigen [51–53] was suggested [11]. According to this hypothesis, podoplanin-ezrin protein complex binds Rho-GDI associated with RhoA-GDP. It causes the release of RhoA-GTP, what allows for its activation by the GDP exchange for GTP. To verify this hypothesis, we analyzed the membranous localization of ezrin and Rho-GDI in our PDPN-positive and PDPN-negative fibroblastic cells. Using confocal microscopy, much higher amounts of both protein associated with membranes were found in podoplanin expressing cells in comparison to control fibroblasts, supporting the existence of mechanism proposed above.

In summary, we have shown that podoplanin increases the motility of fibroblasts and facilitates their interaction with endothelial cells. This, respectively, favors movement of fibroblasts into the breast tumor stroma and affects tumor angiogenesis, what creates a favorable microenvironment for breast cancer progression.

## Supporting information

S1 Fig Characteristics of human fibroblastic cell lines with overexpression of podoplanin. Expression of podoplanin in fibroblastic cell lines transduced with pRRL-CMV-PDPN-IRES-PURO vector containing podoplanin cDNA (MSU1.1 PDPN and Hs578 PDPN) and control cells transduced with vector alone (MSU1.1 NC and Hs578 NC) on the level of mRNA **(A)** and protein **(B)**. Real-time RT-PCR was used to analyze podoplanin mRNA. Podoplanin levels were normalized against *ACTB* gene expression and cell line MSU.1 NC was assigned as a calibrator sample. Results are expressed as mean ±SD. Western blot with anti-podoplanin rabbit polyclonal antibodies was used to analyze podoplanin in cell lysates. **(C)** Binding of anti-podoplanin antibodies to membrane proteins isolated from MSU1.1 PDPN cells. Cell lysates equivalent to 15 μg of protein or 15 μg of membranous proteins were separated by SDS-PAGE under reducing conditions on a 12% gel and electrophoretically transferred onto a nitrocellulose membrane.
(TIF)
