# Supplementary figures and images for: Podoplanin increases the migration of human fibroblasts and affects the endothelial cell network formation: A possible role for cancer-associated fibroblasts in breast cancer progression
Source: PLoS One. 2017 Sep 22;12(9):e0184970. doi: 10.1371/journal.pone.0184970 (PMC5609749; doi:10.1371/journal.pone.0184970)

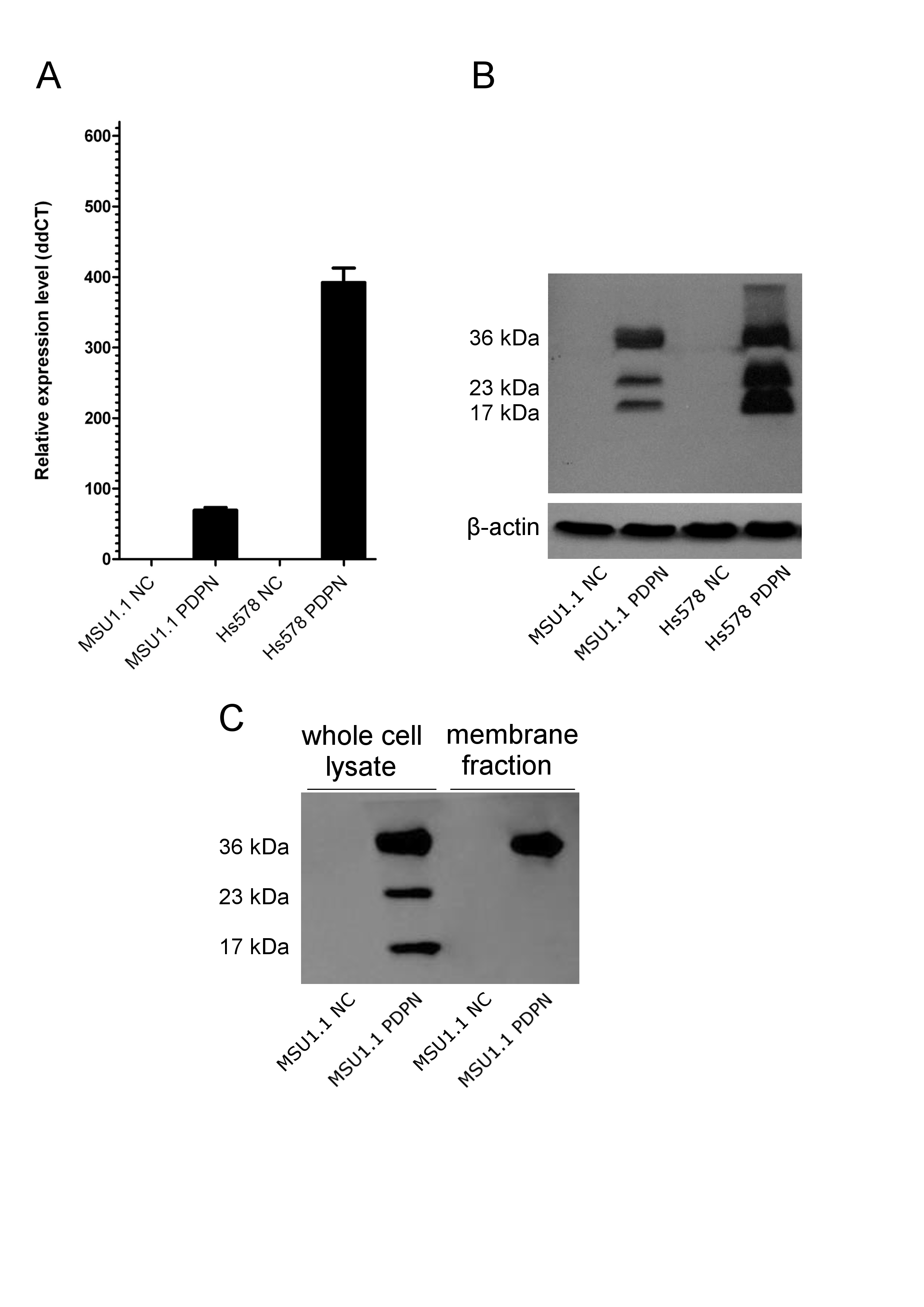

Supplement: S1 Fig — Expression of podoplanin in fibroblastic cell lines transduced with pRRL-CMV-PDPN-IRES-PURO vector containing podoplanin cDNA (MSU1.1 PDPN and Hs578 PDPN) and control cells transduced with vector alone (MSU1.1 NC and Hs578 NC) on the level of mRNA (A) and protein (B). Real-time RT-PCR was used to analyze podoplanin mRNA. Podoplanin levels were normalized against ACTB gene expression and cell line MSU.1 NC was assigned as a calibrator sample. Results are expressed as mean ±SD. Western blot with anti-podoplanin rabbit polyclonal antibodies was used to analyze podoplanin in cell lysates. (C) Binding of anti-podoplanin antibodies to membrane proteins isolated from MSU1.1 PDPN cells. Cell lysates equivalent to 15 μg of protein or 15 μg of membranous proteins were separated by SDS-PAGE under reducing conditions on a 12% gel and electrophoretically transferred onto a nitrocellulose membrane. (TIF) [file pone.0184970.s001.tif]
